# Argonaute-2 autoantibodies: a promising biomarker for predicting mortality in HBV-related acute-on-chronic liver failure patients with cirrhosis

**DOI:** 10.3389/fcimb.2024.1407064

**Published:** 2024-07-25

**Authors:** Yixuan Wang, Yue Hu, Jiaqi Li, Huailu Ma, Zongqi Shi, Chaojing Wen, Yu Long, Ziwei Li, Hang Sun, Yixuan Yang, Xiaofeng Shi

**Affiliations:** ^1^ Department of Infectious Diseases, Key Laboratory of Molecular Biology for Infectious Diseases (Ministry of Education), Institute for Viral Hepatitis, The Second Affiliated Hospital, Chongqing Medical University, Chongqing, China; ^2^ Department of Vascular Surgery, Chongqing Medical University, Chongqing, China; ^3^ Institute of Translational Medicine, Zhejiang University School of Medicine, Zhejiang, Hangzhou, China; ^4^ Central Laboratory, Chongqing University FuLing Hospital, Chongqing, China

**Keywords:** argonaute-2 autoantibodies, chronic hepatitis B, mortality, acute-on-chronic liver failure, predictive biomarker

## Abstract

**Background & aims:**

HBV infection initiates autoimmune responses, leading to autoantibody generation. This research explores the role of autoantibodies in HBV-related Acute-on-Chronic Liver Failure (ACLF), offering novel perspectives for clinical management.

**Method:**

We applied immunoprecipitation and iTRAQ techniques to screen for autoantibodies in serum from HBV-related cirrhosis patients and conducted detection with conformation- stabilizing ELISA in a cohort of 238 HBV-infected individuals and 49 health controls. Our results were validated in a retrospective cohort comprising 106 ACLF patients and further assessed through immunohistochemical analysis in liver tissues from an additional 10 ACLF cases.

**Results:**

Utilizing iTRAQ, we identified Argonaute1-3 autoantibodies (AGO-Abs) in this research. AGO2-Abs notably increased in cirrhosis, decompensation, and further in ACLF, unlike AGO1-Abs and AGO3-Abs. This reflects disease severity correlation. Logistic regression and COX models confirmed AGO2-Abs as independent prognostic indicators for decompensated liver cirrhosis (DLC) and ACLF. In the ROC analysis, AGO2-Abs showed significant diagnostic value for predicting 28- and 90-day mortality (AUROC = 0.853 and 0.854, respectively). Furthermore, combining AGO2-Abs with the Child-Pugh, MELD, and AARC scores significantly improved their predictive accuracy (P < 0.05). Kaplan-Meier analysis showed poorer survival for AGO2-Abs levels above 99.14μg/ml. These findings were supported by a retrospective validation cohort. Additionally, immunohistochemistry revealed band-like AGO2 expression in periportal liver areas, with AGO2-Abs levels correlating with total bilirubin, indicating a potential role in exacerbating liver damage through periportal functions.

**Conclusions:**

AGO2-Abs is a robust biomarker for predicting the mortality of patients with HBV-related ACLF.

## Introduction

Acute-on-chronic liver failure (ACLF) is a clinical syndrome characterized by acute deterioration of liver function, marked by an extremely high short-term mortality rate, presenting significant challenges in disease management. Thus, comprehensive exploration of the pathophysiological characteristics of ACLF and precise evaluation of its prognosis are crucial for enhancing disease management. The Asian Pacific Association for the Study of the Liver (APASL) defines ACLF as an acute exacerbation of liver function in patients with pre-existing liver disease or cirrhosis, highlighting the significance of elevated bilirubin and coagulopathy in the diagnosis of ACLF. This contrasts with the European Foundation for the Study of Chronic Liver Failure (CLIF), which uses systemic organ or system failure as the primary diagnostic criterion (Sarin and Choudhury, 2016; Sarin et al., 2019). In the Asia-Pacific region, the onset and exacerbation of ACLF are closely linked to HBV infection, with chronic hepatitis and cirrhosis caused by HBV being the primary underlying conditions leading to ACLF in this area (Li et al., 2016). The liver pathology of HBV-ACLF patients demonstrates distinctive features, mainly showing as submassive hepatic necrosis with cholestasis and/or canalicular bilirubin stasis (sepsis), in contrast, alcohol-related ACLF patients primarily exhibit inflammation and bilirubin stasis (Mookerjee et al., 2011; Li et al., 2015). Hence, HBV-ACLF warrants distinct consideration and discussion.

Patients with cirrhosis are at a higher risk of developing ACLF compared to those without cirrhosis (Asrani et al., 2013; Hsu et al., 2023). Additionally, HBV infection correlates with the onset of various autoimmune pathologies, such as systemic lupus erythematosus, aplastic anemia, antiphospholipid syndrome, rheumatoid arthritis, type 1 diabetes, multiple sclerosis, and thyroid diseases (Maya et al., 2008). This indicates that HBV contributes to the disordering of the autoimmune system. During HBV infection, the apoptosis and necrosis of infected hepatocytes, driven by T cells and NK cells, alongside mitochondrial dysfunction and hepatic stellate cell activation, result in ongoing chronic inflammation in the liver (Piconese et al., 2018; Loureiro et al., 2023; You et al., 2023). Against the backdrop of autoimmune disarray, this inflammation and the subsequent leakage of cellular proteins can trigger the immune system to generate systemic autoantibodies. Autoantibodies are primarily classified into IgM and IgG types. IgM antibodies are considered to be involved in the clearance of cellular debris and degrade over a short term; IgG antibodies, associated with the breach of central immune tolerance, can persist and proliferate, often correlating with pathological processes (Tsokos et al., 2013). Recent studies on the role of autoantibodies in the diagnosis and progression of tumors and neurological diseases are increasing, confirming their clinical diagnostic value (Ezzikouri et al., 2015; Zhang et al., 2020; Do et al., 2021; Gövert et al., 2023). Hence, we hypothesized that the disruption of the autoimmune system, particularly through cirrhosis, may lead to the production of specific IgG autoantibodies, contributing to the progression of the disease. To validate the clinical utility of these antibodies, this study investigates the role of autoantibodies in HBV-related cirrhotic ACLF from an autoimmune perspective. The aim is to unveil new insights into the mechanisms of this complex disease and to identify potential biomarkers. The approach involves using immunoprecipitation (IP) and Isobaric Tags for Relative and Absolute Quantitation (iTRAQ) technologies to screen for potential autoantibodies in the serum of patients with HBV-related cirrhosis, examining their correlation with the prognosis of ACLF.

## Patients and methods

### Patients

This investigation, approved by the Ethics Review Committee of the Second Affiliated Hospital of Chongqing Medical University, integrates both prospective (exploratory and test cohorts) and retrospective (validation cohort) elements. Recruitment spanned from October 2021 to January 2023, involving 297 chronic Hepatitis B Virus (HBV) infected patients and 72 healthy controls (HC) from the community. Chronic HBV infection diagnosed as per APASL guidelines (Sarin et al., 2016), were stratified into non-cirrhotic chronic Hepatitis B (CHB), compensated liver cirrhosis (CLC), decompensated liver cirrhosis without liver failure (DLC), and acute-on-chronic liver failure (ACLF) based on clinical assessments (Yoshiji et al., 2021). ACLF cases were identified following AARC criteria (Sarin et al., 2019).

Exclusion criteria encompassed pregnant individuals or those who had been pregnant within six months, co-infections (HIV, HCV, HAV, HDV, human cytomegalovirus, Epstein-Barr virus), autoimmune or connective tissue diseases, liver damage from various etiologies, malignant tumors, severe psychiatric conditions impeding follow-up/treatment, and loss to follow-up. During enrollment, comprehensive patient data including medical history, physical examination, and biochemical profiles were collected, followed by the acquisition of survival data through medical records or direct contact six months post-enrollment.

Following exclusion, HC and CHB groups, matched by age and gender with the ACLF, DLC, and CLC groups, comprised 248 chronic HBV infected patients and 59 healthy controls for final analysis. These were divided into an exploratory cohort, including 10 HC and 10 HBV-related cirrhosis patients (details in **Supplementary Table S1**), and a test cohort containing 49 HC and 239 HBV-infected patients: 41 with CHB, 54 with CLC, 40 with DLC, and 103 with ACLF. A retrospective validation cohort comprised 106 HBV-related ACLF patients, admitted between May 2020 and May 2021, aligning diagnostic criteria with the prospective study. Clinical characteristics of the test and validation cohort are available in **Table 1**. Additionally, AGO protein expression in liver tissues from 10 ACLF cases was assessed using paraffin-embedded sections sourced from the Pathology Center of Chongqing Medical University. Informed consent was systematically obtained from all participants, adhering to ethical standards set forth by the review committee.

**Table 1:** Baseline characteristics of the test cohort.

	HC n=49	CHB n=41	CLC n=54	DLC n=40	ACLF n=103	VC n=106
**Years**	**48 (41, 57)**	**49 (39, 54)**	**50 (41, 55)**	**51 (42, 55)**	**51 (40, 59)**	**50 (42, 58)**
**Gender (% male)**	**61.2%**	**63.4%**	**63.0%**	**72.5%**	**75.5%**	**77.4%**
**WBC (count 10^9^/L)**	**5.4(4.7, 6.3)**	**5.5 (4.4, 6.2)**	**4.3(3.6, 5.1)**	**3.4 (2.6, 4.5)**	**5.6 (3.8, 8.6)**	**5.8 (4.5, 7.4)**
**PLT (count 10^9^/L)**	**192 (150, 234)**	**169 (132, 231)**	**102 (86, 13)**	**63 (36, 119)**	**77 (38, 112)**	**82 (52, 100)**
**INR**	**0.97 (0.93, 1.01)**	**0.98 (0.93, 1.04)**	**1.08 (0.99, 1.17)**	**1.39 (1.29, 1.56)**	**2.4 (1.85, 3.08)**	**2.26 (1.76, 2.89)**
**ALB (g/L)**	**42.3 (40.2, 45.4)**	**42.0 (40.0, 44.0)**	**39.0 (36.8, 41.1)**	**35.5 (31.6, 39.4)**	**31.5 (29.2, 34.2)**	**32.5 (29.7, 35.5)**
**ALT (U/L)**	**19 (14, 30)**	**30 (20, 228)**	**34 (25, 62)**	**32 (17, 48)**	**242 (93, 556)**	**211 (80, 430)**
**AST (U/L)**	**24 (20, 32)**	**41 (24, 140)**	**42 (30, 58)**	**48 (32, 74)**	**164 (83, 402)**	**132 (76, 273)**
**TBIL (μmol/L)**	**14.5 (11.7, 20.1)**	**14.8 (10.7,21.9)**	**39.1 (35.2, 47.2)**	**44.1 (35.8, 59.0)**	**267.3 (191.7, 343.6)**	**234.6 (162.3, 322.4)**
**SCR (μmol/L)**	**54.3 (42.5, 65.2)**	**64.4 (54.4, 74.4)**	**68.2 (61.1, 76.7)**	**67.6 (60.4, 81.7)**	**60.8 (51.5, 68.9)**	**63.2 (54.6, 70.5)**
**LAC (mmol/L)**	**-**	**-**	**-**	**2.36 (1.96, 2.68)**	**3.22 (2.65, 3.85)**	**3.40 (2.92, 3.82)**
**HBV DNA(log10 IU/ml)**	**-**	**6.0 (2.4, 8.0)**	**2.0 (2.0, 2.0)**	**2.2(2.0, 3.5)**	**5.6 (2.1, 7.1)**	**5.1 (2.0, 7.1)**
**Child-pugh**	**-**	**-**	**6 (6, 6)**	**8 (6, 8)**	**11 (10, 12)**	**11 (10, 12)**
**MELD**	**-**	**-**	**8.0 (6.9, 9.9)**	**11.5 (9.8, 14.5)**	**22.5 (19.7, 26.4)**	**22.2 (18.6, 25.4)**
**AARC**	**-**	**-**	**-**	**7 (7, 8)**	**3 (2, 3)**	**9 (8, 11)**
**Deceased**	**-**	**-**	**-**	**2 (5.0%)**	**48 (46.6%)**	**45 (42.5%)**
**Hepatic Encephalopathy**	**-**	**-**	**-**	**4 (10.0%)**	**44 (42.7%)**	**43 (40.6%)**
**Peritonitis**	**-**	**-**	**-**	**18 (45.0%)**	**80 (77.7%)**	**83 (78.3%)**
**AGO1-Abs (μg/ml)**	**8.2 (5.5, 24.3)**	**7.1 (5.1, 14.9)**	**11.4 (8.6, 15.7)**	**11 (8.7, 14.4)**	**8.9 (3.5, 18.7)**	**-**
**AGO2-Abs (μg/ml)**	**27.4 (20.5, 32.5)**	**32.1 (20.3, 51.2)**	**39.7 (29.7, 55.2)**	**52.2 (37.1, 79.1)**	**106.6 (57.1, 171.0)**	**98.7 (57.7, 156.9)**
**AGO3-Abs (μg/ml)**	**12.3 (10.5, 23.4)**	**15.3 (11.5, 29.3)**	**19.7 (14.6, 31.6)**	**19.7 (12.2, 24.2)**	**17.5 (13.9, 25.2)**	**-**

VC, validation cohort; ACLF, Acute-on-chronic liver failure; DLC, Decompensated liver cirrhosis without liver failure; CLC, Compensated liver cirrhosis; CHB, Chronic Hepatitis B; HC, Health controls; HE, Hepatic Encephalopathy; PI, Peritonitis; WBC, White Blood Cell count; PLT, Platelet count; INR, International Normalized Ratio; ALB, Albumin; ALT, Alanine Aminotransferase; AST, Aspartate Aminotransferase; TBIL, Total Bilirubin; SCR, Serum Creatinine; LAC, Lactate; MELD, Model for End-Stage Liver Disease Score; AARC, APASL ACLF Research Consortium score.

### Cell culture

HEK293T and HepG2 cells were procured from the American Type Culture Collection (ATCC) and cultured in Dulbecco’s Modified Eagle Medium (DMEM) supplemented with 10% fetal bovine serum (FBS) and 1% penicillin-streptomycin. Cultivation was conducted at 37°C under a 5% CO2 atmosphere.

### Immunoprecipitation and autoantibody profiling

Serum samples from 10 healthy controls and 10 cirrhotic subjects within the exploratory cohort were equitably amalgamated. Subsequently, pooled sera from both cohorts were subjected to Protein G affinity chromatography *(BDTL003, Biodragon)* to isolate serum IgG. These purified IgG were covalently attached to latex beads *(PS0250CLA, Vdobiotech)* using EDAC *(E6383, SIGMA)* cross-linking. The resultant IgG-beads conjugates were incubated overnight with HepG2 cell lysates to perform immunoprecipitation, capturing the relevant antigen-antibody complexes. Following incubation, antigen-antibody complexes are eluted from the beads via denaturation. The complexes are then subjected to peptide-level identification using iTRAQ technology, This process allows for a comparative analysis of antigens differentially bound by IgG in healthy versus cirrhotic conditions, thereby revealing unique autoantibody profiles.

### Plasmids and lentiviruses

The coding sequences of human AGO1, AGO2, and AGO3 were cloned into the GV341 vector with a Flag tag. Standard packaging plasmids and procedures were used to generate lentiviruses for gene expression in 293T cells. Cells were infected with the lentivirus using a standard protocol, and infected cells were selected with 2 μg/ml puromycin for 2 days to establish stable cell lines.

### Cell-based assay and immunoprecipitation

To corroborate the iTRAQ findings, this investigation utilized both cell-based assay (CBA) and immunoprecipitation for the validation of AGO antibodies in serum samples from the exploratory cohort. In the CBA approach, HEK293T-Flag-AGO OV cells, along with control HEK293T cells, were cultured on coverslips, then washed with PBS, incubated in 4% paraformaldehyde for 15 minutes, and permeabilized with 0.5% Triton-X solution at room temperature for 20 minutes. Samples were blocked with 5% goat serum and then incubated with patient serum (*1:100*) and anti-Flag antibody *(1:1000, 20543-1-AP, Proteintech)* for 1 hour, followed by incubation with appropriate secondary antibodies *(1:2000, K1208, K1203, Apexbio)* to elucidate co-localization via fluorescence confocal microscopy.

Simultaneously, samples for protein quantification via CBA assay were subjected to immunoprecipitation: 1 µl test serum was incubated with approximately 3.65mg lysed HepG2 proteins and 20 µl of protein A/G agarose beads *(sc-2003, SANTA CRUZ)*, followed by overnight incubation at 4°C. The beads were then washed, centrifuged, and denaturated to elute bound proteins. The presence and specificity of AGO antibodies were finally confirmed through Western Blotting using anti-AGO1 *(1:1000, #5030, Cell Signaling Technology)*, anti-AGO2 *(1:1000, 67934-1-lg, Proteintech)*, and anti-AGO3 antibodies *(1:1000, 19692-1-AP, Proteintech)*.

### Conformation-stabilizing ELISA

A conformation-stabilizing coating buffer was prepared by augmenting the standard coating buffer *(BF06212, Biodragon)* with 30% glycerol (Moritz et al., 2022). Proteins AGO1, AGO2, and AGO3 *(RP02933, RP01132, RP02965, ABclonal Technology)* were then coated onto ELISA plates at a concentration of 0.3 µg/ml. Test sera, diluted to 1:1000, were incubated overnight at 4°C for antigen-antibody interaction. The bound antibodies were detected using HRP-conjugated secondary antibodies *(1:20000, ZB-2304, ZSGB-BIO)*, and absorbance was measured at an optical density of 450 nm (OD450). The assay included rabbit anti-AGO *(A6022, A6802, A19819, ABclonal Technology)* antibodies as positive controls and used HRP-conjugated goat anti-rabbit IgG *(1:20000, ZB-2301, ZSGB-BIO)* for signal development. ΔOD was calculated by subtracting the blank control’s absorbance from the sample’s absorbance, facilitating the relative quantification of AGO antibodies in the samples.

### Immunohistochemical staining

Paraffin-embedded tissue sections were subjected to sequential dewaxing in xylene, rehydration through a graded ethanol series, and antigen retrieval in citrate buffer (pH 6.0). Endogenous peroxidase activity was quenched with 3% H_2_O_2_, followed by blocking with 5% BSA. Sections were incubated overnight at 4°C with primary antibodies against AGO1, AGO2, and AGO3. HRP-conjugated secondary antibodies were then applied, followed by chromogenic detection with DAB. Sections were counterstained with hematoxylin, dehydrated, and prepared for microscopic evaluation.

### Statistical analysis

Continuous variables were presented as median and interquartile range (Q1-Q3), while categorical data were summarized as frequencies and percentages. Non-normally distributed variables were analyzed using the Kruskal-Wallis non-parametric test. Correlations were assessed using Spearman’s correlation coefficient. Statistical analyses were conducted using GraphPad Prism and SPSS software. The factors affecting survival rates were evaluated using binary logistic regression and Cox proportional hazards regression models. Receiver operating characteristic (ROC) curve analysis was performed with MedCalc software to evaluate the predictive performance of prognostic variables. Kaplan-Meier survival analysis was employed to assess the impact of prognostic variables on mortality, with survival curves plotted using GraphPad Prism.

## Results

### ITRAQ identification results

**Figure 1:**
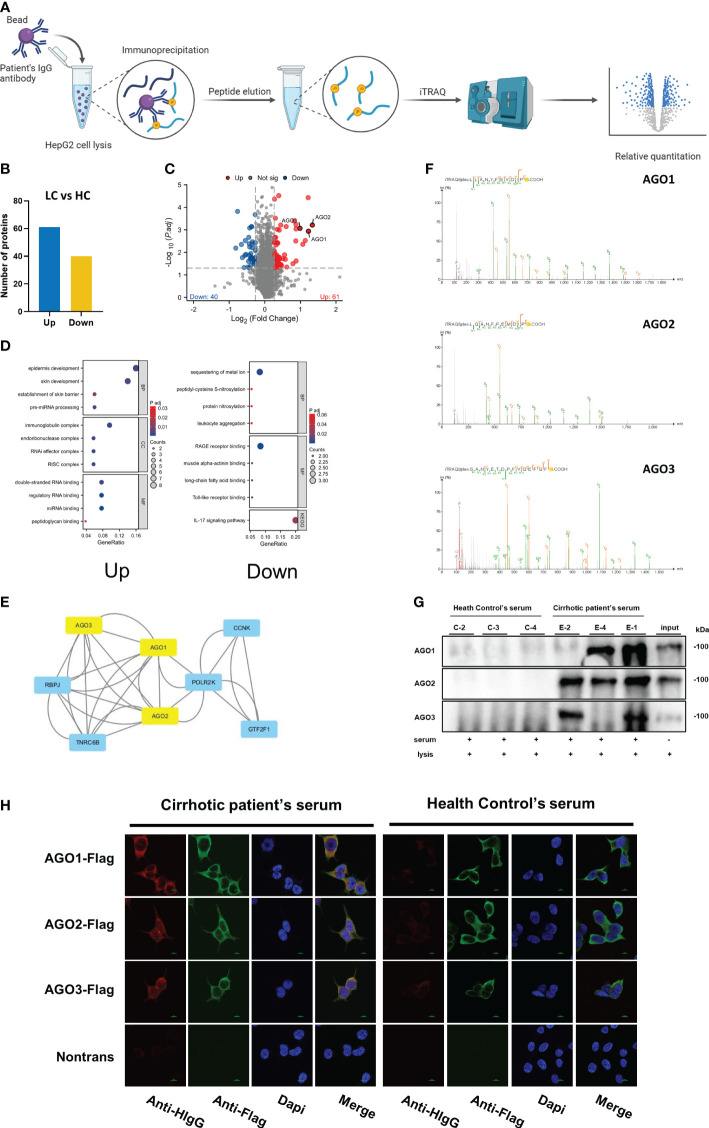
Identification of Differential Capture Antigens Associated with Cirrhosis: **(A)** Schematic illustration of the workflow for identifying differential capture antigens. Created with BioRender.com. **(B)** column presenting differentially identified antigens by iTRAQ; **(C)** Volcano plot illustrating differentially identified antigens by iTRAQ; **(D)** Functional enrichment analysis of differentially identified proteins by iTRAQ; **(E)** Hub gene analysis of differentially identified proteins by iTRAQ; **(F)** Mass spectrometry secondary spectra for AGO1, AGO2, AGO3; **(G)** Validation of AGO-Abs in serum by immunoprecipitation; **(H)** Validation of AGO-Abs in serum by cell-based assay.

Utilizing iTRAQ on samples of ‘IgG captured proteins’ from the exploratory cohort comprising HC and cirrhotic patients (**Figure 1A**), our research discerned a variety of proteins with differential IgG interactions, which led to insights into the diversity of antibodies in patient sera. We successfully identified 60 proteins exhibiting increased IgG binding and 40 with reduced binding (fold change >1.2, as shown in **Figures 1B, C**). The functionally enriched analysis of these proteins indicated that those with augmented IgG interaction predominantly involved pathways related to RNA binding, processing, and degradation. In contrast, proteins with diminished IgG binding were primarily associated with metal ion sequestration, RAGE receptor binding, and the IL-17 signaling pathway (**Figure 1D**). Through a focused hub gene analysis, the Argonaute (AGO) proteins, comprising AGO1, AGO2, and AGO3, was identified as significantly linked to liver cirrhosis (refer to **Figures 1E, F**), underscoring the importance of anti-Argonaute autoantibodies (AGO-Abs). To corroborate these findings, IP and CBA techniques were applied to the serum of the exploratory cohort, confirming the authenticity of the detected AGO-Abs (**Figures 1G, H**).

### AGO2 autoantibodies is associated with the severity of HBV related liver disease

**Figure 2:**
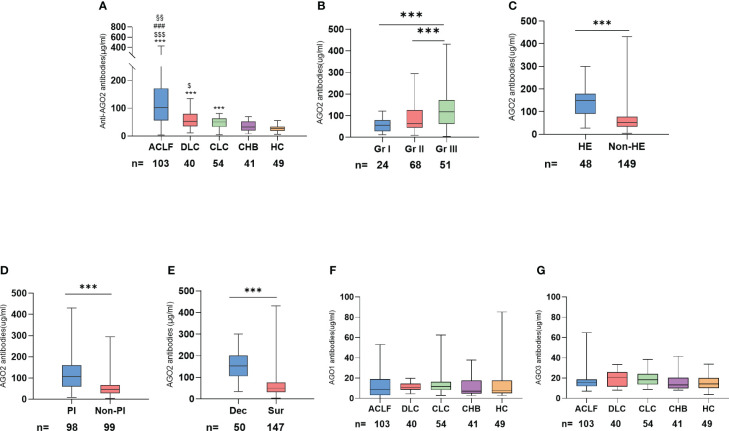
Relationship between AGO-Abs levels and severity of liver disease in the test cohort: **(A)** Distribution of AGO2-Abs levels across different severities of liver disease (*p <0.05, **p <0.01, ***p <0.001 vs HC; $ vs CHB; # vs CLC; § vs DLC); **(B)** Comparison of AGO2-Abs in DLD and ACLF patients across different AARC score grades; **(C)** Comparison of AGO2-Abs levels in cirrhotic patients with and without hepatic encephalopathy; **(D)** Comparison of AGO2-Abs levels in cirrhotic patients with and without peritonitis; **(E)** Comparison of AGO2-Abs between deceased and surviving patients with cirrhosis; **(F)** Distribution of AGO1-Abs levels across different severities of liver disease; **(G)** Distribution of AGO3-Abs levels across different severities of liver disease. ACLF, Acute-on-chronic liver failure; DLC, Decompensated liver cirrhosis without liver failure; CLC, Compensated liver cirrhosis; CHB, Chronic Hepatitis B; HC, Health controls; HE, Hepatic Encephalopathy; PI, Peritonitis; Dec, Deceased; Sur, Survived; Gr I, AARC 5-7; Gr II, AARC 8-10; Gr III, AARC 11-15. $ p <0.05, §§ p <0.01, ***/$$$/### p <0.001; * vs HC, $ vs CHB, # vs CLC, § vs DLC.

Our comprehensive analysis of a test cohort with 238 patients, including 103 with ACLF, 40 with DLC, 54 with CLC, 41 with CHB, and 49 healthy controls, revealed significant variations in AGO2-Abs levels across different stages of HBV infection. The median levels of AGO2-Abs were notably lower in HC and non-cirrhotic CHB patients, increasing with the disease progression to cirrhosis and decompensation, and further elevating in ACLF (**Table 1**; **Figure 2A**). Furthermore, when ACLF and DLC patients were stratified by AARC scores, grade 3 patients exhibited significantly higher AGO2-Abs levels compared to grade 1 and 2 (**Figure 2B**). Notably, in the ACLF/DLC group, patients with severe complications (e.g., hepatic encephalopathy, spontaneous bacterial peritonitis) and deceased patients had significantly higher AGO2-Abs levels than those without these conditions (**Figures 2C–E**). In contrast, the levels of AGO1-Abs and AGO3-Abs did not demonstrate similar trends related to disease severity (**Figures 2F, G**). Additionally, levels of autoantibodies for AGO1, AGO2, and AGO3 showed no significant correlation with circulating HBV DNA load.

The results above suggest that AGO2-Abs levels are associated with the severity of HBV-related cirrhosis, increasing as liver damage progresses. Therefore, AGO2-Abs emerged as the focal point of our study.

### AGO2 autoantibodies predicts mortality in DLC/ACLF patients

#### Analysis of the test cohort

A total of 143 patients from the test cohort were analyzed, consisting of 40 with DLC and 103 with ACLF. During a 180-day follow-up, 50 patients (27.78%) deceased, with ACLF mortality significantly higher than DLC (46.6% vs 5%, P<0.001). For the combined DLC/ACLF subgroup (n=143), the mortality rates at 28 days and 90 days were observed to be 12.22% (22 cases) and 26.67% (48 cases), respectively.

**Figure 3:**
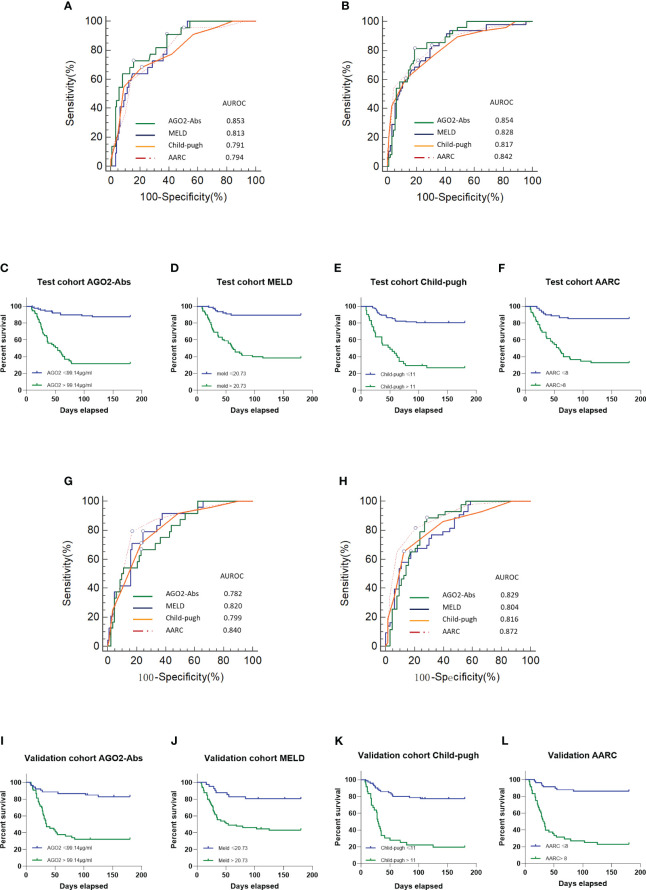
Mortality Prediction and Survival Analysis: **(A)** Prediction of 28-day mortality for DLC/ACLF patients in the test cohort; **(B)** Prediction of 90-day mortality for DLC/ACLF patients in the test cohort; **(C)** DLC/ACLF patients in the test cohort with AGO2-Abs > 99.14μg/ml exhibit significantly lower survival rates; **(D)** DLC/ACLF patients in the test cohort with MELD > 20.73 have significantly lower survival; **(E)** DLC/ACLF patients in the test cohort with Child-Pugh > 11 exhibit significantly lower survival rates; **(F)** DLC/ACLF patients in the test cohort with AARC > 8 demonstrate significantly lower survival rates; **(G)** Prediction of 28-day mortality for ACLF patients in the validation cohort; **(H)** Prediction of 90-day mortality for ACLF patients in the validation cohort. **(I)** Patients in validation cohort with AGO2-Abs > 99.14μg/ml show significantly lower survival rates; **(J)** Patients in validation cohort with MELD > 20.73 have significantly lower survival; **(K)** Patients in validation cohort with Child-Pugh > 11 exhibit significantly lower survival rates; **(L)** Patients in validation cohort with AARC > 8 demonstrate significantly lower survival rates.

The impact of AGO2-Abs on mortality endpoints and its predictive value for 28-day and 90-day mortality rates were assessed using binary logistic regression and Cox proportional hazards regression models. In both univariate analyses where AGO2-Abs was assessed alone, and multivariate analyses incorporating existing scoring systems such as MELD, Child-Pugh, and AARC, the regression models demonstrated P-values less than 0.05. Thus, AGO2-Abs was identified as an independent predictor of mortality. It exhibited significant predictive value for 28-day and 90-day mortality rates in univariate analysis. Even after integrating existing scoring systems into the multivariate models, AGO2-Abs remained an independent prognostic factor (P<0.05, **Tables 2**, **3**). Subsequent ROC analysis indicated that AGO2-Abs exhibited high diagnostic accuracy in predicting 28-day and 90-day mortality rates (AUROC = 0.853 and 0.854, respectively) in patients with DLC/ACLF, paralleling the diagnostic performance of MELD, Child-Pugh and AARC scores (**Table 4**; **Figures 3A, B**). Notably, the incorporation of AGO2-Abs into the combined ROC diagnostic model with MELD, Child-Pugh and AARC scores significantly enhanced diagnostic accuracy (**Table 4**).

**Table 2:** Logistic regression analysis of predictive factors for mortality in DLC and ACLF patients.

	odd ratios	95%CI	P value	R²
Univariate				
**AGO2-Abs**	**1.017**	**1.010~1.023**	**<0.001**	**0.316**
**Child-pugh**	**1.995**	**1.552~2.565**	**<0.001**	**0.390**
**MELD**	**1.290**	**1.178~1.412**	**<0.001**	**0.435**
**AARC**	**2.656**	**1.919~3.675**	**<0.001**	**0.478**
Add to Child-pugh				
**AGO2-Abs**	**1.011**	**1.004~1.018**	**0.001**	
**Child-pugh**	**1.753**	**1.346~2.282**	**<0.001**	
**Combined**			**<0.001**	**0.475**
Add to MELD				
**AGO2-Abs**	**1.011**	**1.004~1.018**	**0.002**	
**MELD**	**1.236**	**1.125~1.375**	**<0.001**	
**Combined**			**<0.001**	**0.507**
Add to AARC				
**AGO2-Abs**	**1.011**	**1.004~1.018**	**0.003**	
**AARC**	**2.304**	**1.646~3.225**	**<0.001**	
**Combined**			**<0.001**	**0.542**

**Table 3:** Cox proportional hazards model analysis of Short-term mortality predictive factors in DLC and ACLF.

	Hazard ratios	95%CI	-2 Log Likelihood	P value
28-day mortality				
Univariate				
**AGO2-Abs**	**1.010**	**1.006~1.013**	**192.754**	**<0.001**
**Child-pugh**	**1.707**	**1.345~2.167**	**191.342**	**<0.001**
**MELD**	**1.115**	**1.063~1.169**	**198.248**	**<0.001**
**AARC**	**1.684**	**1.347~2.105**	**193.792**	**<0.001**
Add to Child-pugh				
**AGO2-Abs**	**1.009**	**1.005~1.013**		**<0.001**
**Child-pugh**	**1.620**	**1.243~2.110**		**<0.001**
**Combined**			**177.977**	**<0.001**
Add to MELD				
**AGO2-Abs**	**1.008**	**1.004~1.012**		**<0.001**
**MELD**	**1.090**	**1.032~1.152**		**0.002**
**Combined**			**184.77**	**<0.001**
Add to AARC				
**AGO2-Abs**	**1.008**	**1.004~1.013**		**<0.001**
**AARC**	**1.470**	**1.167~1.852**		**0.001**
**Combined**			**181.797**	**<0.001**
90-day mortality				
Univariate				
**AGO2-Abs**	**1.008**	**1.006~1.011**	**426.079**	**<0.001**
**Child-pugh**	**1.662**	**1.417~1.950**	**412.301**	**<0.001**
**MELD**	**1.117**	**1.081~1.154**	**422.097**	**<0.001**
**AARC**	**1.768**	**1.515~2.065**	**406.681**	**<0.001**
Add to Child-pugh				
**AGO2-Abs**	**1.007**	**1.004~1.009**		**<0.001**
**Child-pugh**	**1.566**	**1.322~1.854**		**<0.001**
**Combined**			**395.567**	**<0.001**
Add to MELD				
**AGO2-Abs**	**1.007**	**1.004~1.009**		**<0.001**
**MELD**	**1.099**	**1.061~1.139**		**<0.001**
**Combined**			**403.871**	**<0.001**
Add to AARC				
**AGO2-Abs**	**1.007**	**1.004~1.010**		**<0.001**
**AARC**	**1.628**	**1.389~1.909**		**0.001**
**Combined**			**380.187**	**<0.001**

**Table 4:** ROC analysis of the predictive efficacy for mortality using AGO2-Abs, Child-Pugh, MELD, and AARC scoring systems.

DLC/ACLF cohort (n=143)						
	28-day mortality			90-day mortality		
	Univariate AUROC	AUROC with AGO2-Abs Combined	P value	Univariate AUROC	AUROC with AGO2-Abs Combined	P value
**AGO2-Abs**	**0.853(0.785-0.907)**	**-**	**-**	**0.854(0.786-0.908)**	**-**	**-**
**MELD**	**0.813(0.739-0.873)**	**0.876(0.811-0.925)**	**0.0505**	**0.828(0.756-0.886)**	**0.878(0.813-0.927)**	**0.0148**
**Child-pugh**	**0.791(0.715-0.854)**	**0.874(0.808-0.924)**	**0.0106**	**0.817(0.743-0.876)**	**0.875(0.809-0.924)**	**0.0062**
**AARC**	**0.794(0.719-0.857)**	**0.874(0.808-0.923)**	**0.0238**	**0.842(0.772-0.898)**	**0.883(0.819-0.931)**	**0.0412**
Validation cohort (n=106)						
**AGO2-Abs**	**0.782(0.691-0.856)**	**-**	**-**	**0.829(0.744-0.896)**	**-**	**-**
**MELD**	**0.820(0.734-0.888)**	**0.858(0.776-0.918)**	**0.1902**	**0.804(0.716-0.875)**	**0.867(0.787-0.925)**	**0.0327**
**Child-pugh**	**0.799(0.710-0.870)**	**0.852(0.769-0.913)**	**0.0984**	**0.816(0.729-0.885)**	**0.866(0.786-0.924)**	**0.0656**
**AARC**	**0.840(0.756-0.904)**	**0.876(0.797-0.932)**	**0.0352**	**0.872(0.794-0.929)**	**0.905(0.832-0.953)**	**0.1073**

The optimal cut-off values for predicting mortality, determined by the Youden index, were found to be >146.54μg/ml for AGO2-Abs, >11 for Child-Pugh, >20.85 for MELD, and >9 for AARC at 28 days. While >99.14μg/ml for AGO2-Abs, >11 for Child-Pugh, >20.73 for MELD, and >8 for AARC at 90 days. Notably, a gradient in the predictive cut-off values for AGO2-Abs was observed for 28-day and 90-day mortality, which was less apparent in MELD, Child-Pugh and AARC scores.

In the Kaplan-Meier survival analysis, stratification of patients at baseline using the optimal cut-off values for 90-day mortality revealed a significantly lower survival rate in patients exceeding the AGO2-Abs (>99.14μg/ml) threshold (**Figure 3C**). Similarly, higher MELD, Child-Pugh, and AARC scores were also associated with lower survival rates (**Figures 3D–F**), indicating that the predictive value of AGO2-Abs for mortality is comparable to that of MELD, Child-Pugh and AARC scores, underscoring its potential clinical utility.

#### Analysis of the validation cohort

We analyzed a validation cohort consisting of 106 patients with ACLF. In the ROC analysis conducted, AGO2-Abs demonstrated high predictive value for 28-day and 90-day mortality rates, comparable to those observed in the test cohort (**Table 4**; **Figures 3G, H**). Moreover, this predictive efficacy was found to be similar to that of Child-Pugh, MELD and AARC scores. Utilizing the predictive cut-off values identified in the test cohort for patient stratification revealed that patients with AGO2-Abs levels exceeding 99.14μg/ml had significantly lower survival rates (**Figures 3I–L**). This finding further confirms the importance of AGO2-Abs as a mortality predictor, with its predictive value being on par with that of MELD, Child-Pugh and AARC scores.

### AGO2 exhibits concentrated expression in the periportal areas

**Figure 4:**
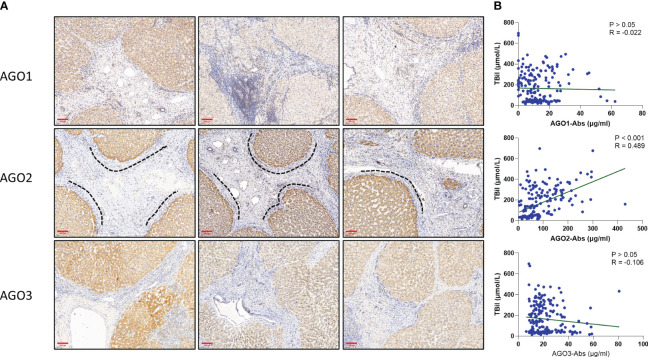
AGO2 and Its Autoantibodies May Influence Periportal Area Functionality: **(A)** AGO2 protein shows band-like concentrated expression in hepatocytes around the periportal area (highlighted by dashed lines), unlike AGO1 and AGO3; **(B)** Correlation between AGO-Abs and total bilirubin (TBil) levels in patients with cirrhosis, cases from the test cohorts comprising CLC, DLC, and ACLF (n=197, P<0.001, R=0.489). Scale bar in immunohistochemistry images=100μm.

In the study of liver tissues from an external 10 patients with ACLF, we conducted immunohistochemical staining to examine the expression of AGO1, AGO2, and AGO3. It was observed that AGO2 demonstrated band-like concentrated expression in hepatocytes around the periportal areas, unlike AGO1 and AGO3 (**Figure 4A**). Further analysis on cirrhosis patients within the test cohort, including CLC, DLC, and ACLF subgroup (n=197), revealed a significant correlation between AGO2-Abs levels and total bilirubin (TBil) levels (P<0.001, R=0.489). No significant correlations were observed between TBil levels and either AGO1-Abs (P>0.05, R=-0.022) or AGO3-Abs (P>0.05, R=-0.106) (**Figure 4B**). This finding suggests that AGO2 may have a role in affecting the function of the periportal area in the liver.

## Discussion

Human AGO proteins, especially AGO2, are central to the RNA silencing complex, playing key roles in gene regulation and cellular processes like apoptosis and differentiation (Jo et al., 2015). AGO2, highly expressed in various cells (Fagerberg et al., 2014), has unique endonuclease activity (Meister et al., 2004; Cheloufi et al., 2010) and significantly impacts immune responses and inflammation (Höck and Meister, 2008; Nakanishi, 2022). In viral hepatitis, AGO2 enhances HBV replication by interacting with miR-146a (Ji et al., 2020) and aids HCV RNA stability through miR-122 (Shimakami et al., 2012). AGO2-linked miRISC complexes help stabilize miRNAs, providing diagnostic insights detectable by anti-AGO2 antibodies (Geekiyanage et al., 2020). This underscores the potential of circulating AGO2-Abs as biomarkers for disease diagnostics.

In this study we explored autoimmune responses by employing IP combined with iTRAQ, identifying a spectrum of proteins. Subsequent bioinformatics analyses honed in on the autoantigens AGO1-3. Utilizing CBA and IP, we confirmed the genuine presence of corresponding AGO1-3 autoantibodies in serum. CBA revealed positive AGO1-3 antibodies in the mixed serum of the exploratory cohort. IP assays indicated AGO2-Abs were consistently present, whereas AGO1-Abs and AGO3-Abs were intermittently undetected due to their low abundance or the sensitivity limits of the assays. Quantitative analyses in the test cohort specifically revealed that AGO2-Abs levels were significantly associated with disease progression, unlike AGO1-Abs and AGO3-Abs. This phenomenon is likely a result of intermolecular epitope spreading, caused by the high degree of homology (approximately 80%) among AGO proteins (Jakymiw et al., 2006), leading to a low-level antibody response to AGO1 and AGO3 in patient serum. Further analysis indicated that AGO2-Abs were pinpointed as not only markers of liver function deterioration but also as independent predictors for short-term mortality in ACLF, highlighting their crucial prognostic value.

Current scoring systems like Child-Pugh, MELD, and AARC are widely used for mortality and disease progression predictions in liver cirrhosis patients (Choudhury et al., 2017). However, these scores rely on variables such as biochemical markers, coagulation profiles, and clinical complications, making them vulnerable to fluctuations caused by symptomatic treatments like albumin supplementation, bilirubin reduction, and ascites management. These interventions may temporarily modify clinical parameters but typically do not change outcomes, underscoring the necessity for stable biomarkers in patient evaluation.

Autoantibody levels, unaffected by these symptomatic treatments, provide such stability. Our analysis showed that AGO2-Abs have similar predictive efficacy to traditional scores in assessing short-term mortality in ACLF. We observed a distinct gradient between optimal cutoff values for 28-day and 90-day mortality (146.54μg/ml vs 99.14μg/ml), not observed in the Child-Pugh, MELD, and AARC systems. This indicates a strong link between AGO2-Abs levels and the progression of ACLF, reflecting their potential for enhancing clinical management precision. Moreover, the incorporation of AGO2-Abs into these traditional scoring systems significantly increased diagnostic accuracy (**Table 4**). This enhancement is likely tied to the symptomatic treatments received before hospital admission, which, while altering biochemical indicators, do not substantially affect overall inflammation or prognosis. Therefore, using AGO2-Abs, which remain stable despite symptomatic treatment, alongside these scores yields a more accurate prediction of mortality, proving advantageous for early prognostic assessments.

Furthermore, we observed significantly elevated AGO2-Abs levels in patients with hepatic encephalopathy (HE). While current theories like ammonia toxicity and neurotransmitter imbalances offer partial explanations for HE’s pathogenesis, they fall short of a comprehensive understanding, with evidence suggesting an autoimmune imbalance connection (Manzhalii et al., 2022). AGO2-Abs, previously detected in systemic autoimmune conditions and chronic Hepatitis C without specific disease association (Vázquez-Del Mercado et al., 2010; Satoh et al., 2013), have recently been found in autoimmune neurological disorder patients, indicating their potential as markers of neurological autoimmunity (Do et al., 2021). This association prompts speculation on the role of AGO2-Abs in HE development, highlighting the need for further investigation to validate their diagnostic utility for HE and to clarify AGO2’s involvement in HE pathogenesis.

We conducted preliminary investigations into the mechanistic link between AGO2 and the deterioration of liver function, identifying a band-like concentration of AGO2 expression around the periportal areas of the liver tissue. This pattern may lead to increased deposition of antigen-antibody complexes in the region, affecting the functionality of the periportal area, contributing to disturbances in hepatic microcirculation and bilirubin excretion, potentially exacerbating liver damage under a cirrhosis backdrop. Additionally, the significant correlation between TBIL levels and AGO2-Abs suggests a potential relationship between AGO2-Abs and bilirubin excretion. However, the specific mechanisms of injury require further study for validation.

Although AGO2-Abs shows promise as a biomarker for predicting mortality in ACLF patients, its utility is constrained due to its production by immune cells, making it less suitable for those on long-term corticosteroids and immunosuppressants, unlike the Child-Pugh, MELD, and AARC scoring systems. This study, limited to a single center, focused on HBV-associated ACLF, omitting an examination of AGO2-Abs in liver failures from diverse etiologies, thereby narrowing its scope. Given that autoimmune activation is prevalent in many forms of liver failure (Bernal et al., 2007), the prognostic potential of AGO2-Abs may extend to other liver failures, the prognostic potential of AGO2-Abs may extend to other liver failures, including those caused by HCV, drug-induced factors, or autoimmune liver diseases. However, due to the use of corticosteroids and immunosuppressants in patients with drug-induced liver injury and autoimmune liver diseases, the accuracy of AGO2-Abs in predicting the prognosis of these types of liver failure may not be as reliable as traditional scoring systems. Future studies should employ a multicentric approach to broaden the scope and enhance the generalizability of findings. Moreover, further research into the mechanistic role of AGO2 and its autoantibodies in liver disease is imperative to enhance our understanding of disease progression and facilitate the development of novel therapeutic strategies.

## Conclusion

In patients with HBV-related cirrhotic ACLF, the presence of AGO2-Abs significantly predicts short-term mortality independently. Integrating AGO2-Abs with established scoring systems like Child-Pugh, MELD, and AARC substantially improves the predictive accuracy for short-term mortality. Hence, AGO2-Abs not only affirm their role as promising biomarkers but also introduce an innovative strategy to refine the management precision of ACLF patients.

## Data availability statement

The raw data supporting the conclusions of this article will be made available by the authors, without undue reservation.

## Ethics statement

The studies involving humans were approved by Ethics Committee of the Second Affiliated Hospital of Chongqing Medical University. The studies were conducted in accordance with the local legislation and institutional requirements. The participants provided their written informed consent to participate in this study.

## Author contributions

YW: Investigation, Data curation, Formal analysis, Writing – original draft, Writing – review & editing. YH: Investigation, Writing – original draft. JL: Investigation, Writing – original draft. HM: Validation, Visualization, Writing – original draft. ZS: Visualization, Writing – original draft. CW: Resources, Writing – original draft. YL: Resources, Writing – original draft. ZL: Resources, Writing – original draft. HS: Writing – review & editing. YY: Project administration, Methodology, Writing – review & editing. XS: Conceptualization, Funding acquisition, Resources, Supervision, Writing – review & editing.
