# Supplementary material for: Argonaute-2 autoantibodies: a promising biomarker for predicting mortality in HBV-related acute-on-chronic liver failure patients with cirrhosis
Source: Front Cell Infect Microbiol. 2024 Jul 25;14:1407064. doi: 10.3389/fcimb.2024.1407064 (PMC11306186; doi:10.3389/fcimb.2024.1407064)
Supplement: Supplementary file 1 [file Table_1.docx]

**Argonaute-2 Autoantibodies:** **A Promising Biomarker for Predicting Mortality in** **HBV Associated Acute-on-Chronic Liver Failure** **Patients with Cirrhosis**

**Supplementary**

***Exploratory cohort***

The exploratory cohort consists of 10 healthy controls and 10 patients with HBV-related cirrhosis, including 5 with compensated cirrhosis and 5 with decompensated cirrhosis. Their specific characteristics are detailed in Table S1.

Table S1. Baseline Characteristics of Patients in the Exploratory cohort.

| **Patient** | **Gender， Years** | **Primary Diagnosis** | **Complications** | **ALB** | **ALT** | **AST** | **TBIL** | **INR** | **LAC** | **SCR** | **WBC** | **PLT** | **HBV DNA (log10 IU/ml)** | **AGO1-Abs  (μg/ml)** | **AGO2-Abs  (μg/ml)** | **AGO3-Abs  (μg/ml)** |
| --- | --- | --- | --- | --- | --- | --- | --- | --- | --- | --- | --- | --- | --- | --- | --- | --- |
| **E-1** | **M, 66** | **DLC** | **ASC, HE** | **34** | **454** | **243** | **73.3** | **1.38** | **2.12** | **64** | **3.81** | **98** | **3.83** | **191.59** | **448.86** | **70.20** |
| **E-2** | **F, 42** | **DLC** | **ASC, PI** | **33.1** | **114** | **65** | **61.3** | **1.36** | **1.89** | **57.9** | **5.22** | **107** | **2.00** | **11.79** | **119.52** | **83.91** |
| **E-3** | **M, 63** | **DLC** | **GIB** | **38.8** | **116** | **52** | **49.2** | **1.17** | **1.78** | **39.5** | **4.85** | **104** | **2.00** | **11.20** | **112.30** | **16.52** |
| **E-4** | **F, 56** | **DLC** | **GIB, ASC, PI** | **37** | **133** | **134** | **64.3** | **1.32** | **2.37** | **51.3** | **4.74** | **168** | **2.29** | **64.69** | **281.34** | **13.52** |
| **E-5** | **F, 45** | **DLC** | **ASC** | **42.2** | **58** | **43** | **39** | **1.29** | **1.94** | **61.9** | **4.1** | **146** | **2.20** | **5.52** | **105.95** | **12.27** |
| **E-6** | **F, 50** | **CLC** | **-** | **40.6** | **37** | **22** | **39.2** | **1.04** | **1.01** | **68** | **4.46** | **133** | **2.23** | **21.54** | **39.35** | **36.46** |
| **E-7** | **M, 48** | **CLC** | **-** | **41.8** | **66** | **23** | **47.5** | **1.16** | **1.42** | **59.7** | **5.64** | **150** | **2.00** | **25.41** | **52.22** | **15.15** |
| **E-8** | **M, 57** | **CLC** | **-** | **45.6** | **40** | **29** | **37** | **0.99** | **0.98** | **54.6** | **3.71** | **100** | **2.00** | **17.81** | **32.67** | **23.06** |
| **E-9** | **F, 43** | **CLC** | **-** | **46.1** | **35** | **24** | **32.8** | **0.98** | **1.13** | **54.6** | **5.45** | **216** | **2.00** | **9.79** | **7.31** | **15.61** |
| **E-10** | **F, 55** | **CLC** | **-** | **43.3** | **32** | **27** | **34.4** | **0.96** | **0.94** | **61.9** | **4.71** | **104** | **2.00** | **11.42** | **25.50** | **13.67** |
| **C-1** | **F, 48** | **HC** | **-** | **45.4** | **9** | **21** | **12.5** | **0.99** | **-** | **54.6** | **6.41** | **132** | **-** | **10.31** | **26.56** | **40.00** |
| **C-2** | **F, 66** | **HC** | **-** | **42.6** | **9** | **35** | **40.8** | **1.03** | **-** | **68** | **4.78** | **150** | **-** | **1.32** | **20.81** | **18.39** |
| **C-3** | **M, 46** | **HC** | **-** | **51.1** | **18** | **21** | **8.8** | **0.91** | **-** | **44.1** | **5.18** | **251** | **-** | **4.50** | **29.20** | **17.49** |
| **C-4** | **F, 56** | **HC** | **-** | **44.4** | **14** | **12** | **15.1** | **0.96** | **-** | **53.4** | **5.68** | **326** | **-** | **8.09** | **43.05** | **17.29** |
| **C-5** | **F, 42** | **HC** | **-** | **41.9** | **13** | **56** | **11.9** | **0.98** | **-** | **43.2** | **4.23** | **153** | **-** | **2.89** | **49.04** | **15.31** |
| **C-6** | **M, 71** | **HC** | **-** | **41.8** | **19** | **25** | **7.5** | **0.94** | **-** | **35.4** | **5.64** | **203** | **-** | **6.50** | **31.71** | **21.61** |
| **C-7** | **F, 47** | **HC** | **-** | **41.3** | **11** | **22** | **19.7** | **0.94** | **-** | **38** | **3.57** | **225** | **-** | **25.43** | **25.54** | **11.87** |
| **C-8** | **M, 56** | **HC** | **-** | **43.1** | **17** | **16** | **5.6** | **1.04** | **-** | **52.5** | **5.14** | **178** | **-** | **30.72** | **30.56** | **11.19** |
| **C-9** | **F, 47** | **HC** | **-** | **47.1** | **35** | **25** | **16.3** | **1.02** | **-** | **48.3** | **6.05** | **241** | **-** | **2.23** | **42.71** | **17.10** |
| **C-10** | **M, 44** | **HC** | **-** | **39.8** | **19** | **17** | **18.5** | **0.99** | **-** | **46.5** | **3.45** | **210** | **-** | **1.49** | **6.40** | **9.12** |

Gender: M = Male, F = Female, ASC: Ascites, HE: Hepatic Encephalopathy, PI: Peritonitis, GIB: Gastrointestinal Bleeding.

***Overexpression of Argonaute protein (AGO) sequences***

*AGO1:* TCTAGAGGATCCAACTTTGTGCCAACCGGTCGCCACCATGGAAGCGGGACCCTCGGGAGCAGCTGCGGGCGCTTACCTGCCCCCCCTGCAGCAGGTGTTCCAGGCACCTCGCCGGCCTGGCATTGGCACTGTGGGGAAACCAATCAAGCTCCTGGCCAATTACTTTGAGGTGGACATCCCTAAGATCGACGTGTACCACTACGAGGTGGACATCAAGCCGGATAAGTGTCCCCGTAGAGTCAACCGGGAAGTGGTGGAATACATGGTCCAGCATTTCAAGCCTCAGATCTTTGGTGATCGCAAGCCTGTGTATGATGGAAAGAAGAACATTTACACTGTCACAGCACTGCCCATTGGCAACGAACGGGTCGACTTTGAGGTGACAATCCCTGGGGAAGGGAAGGATCGAATCTTTAAGGTCTCCATCAAGTGGCTAGCCATTGTGAGCTGGCGAATGCTGCATGAGGCCCTGGTCAGCGGCCAGATCCCTGTTCCCTTGGAGTCTGTGCAAGCCCTGGATGTGGCCATGAGGCACCTGGCATCCATGAGGTACACCCCTGTGGGCCGCTCCTTCTTCTCACCGCCTGAGGGCTACTACCACCCGCTGGGGGGTGGGCGCGAGGTCTGGTTCGGCTTTCACCAGTCTGTGCGCCCTGCCATGTGGAAGATGATGCTCAACATTGATGTCTCAGCCACTGCCTTTTATAAGGCACAGCCAGTGATTGAGTTCATGTGTGAGGTGCTGGACATCAGGAACATAGATGAGCAGCCCAAGCCCCTCACGGACTCTCAGCGCGTTCGCTTCACCAAGGAGATCAAGGGCCTGAAGGTGGAAGTCACCCACTGTGGACAGATGAAGAGGAAGTACCGCGTGTGTAATGTTACCCGTCGCCCTGCTAGCCATCAGACATTCCCCTTACAGCTGGAGAGTGGACAGACTGTGGAGTGCACAGTGGCACAGTATTTCAAGCAGAAATATAACCTTCAGCTCAAGTATCCCCATCTGCCCTGCCTACAAGTTGGCCAGGAACAAAAGCATACCTACCTTCCCCTAGAGGTCTGTAACATTGTGGCTGGGCAGCGCTGTATTAAAAAGCTGACCGACAACCAGACCTCGACCATGATAAAGGCCACAGCTAGATCCGCTCCAGACAGACAGGAGGAGATCAGTCGCCTGATGAAGAATGCCAGCTACAACTTAGATCCCTACATCCAGGAATTTGGGATCAAAGTGAAGGATGACATGACGGAGGTGACAGGGCGAGTGCTGCCGGCGCCCATCTTGCAGTACGGCGGCCGGAACCGGGCCATTGCCACACCCAATCAGGGTGTCTGGGACATGCGGGGGAAACAGTTCTACAATGGGATTGAGATCAAAGTCTGGGCCATCGCCTGCTTCGCACCCCAAAAACAGTGTCGAGAAGAGGTGCTCAAGAACTTCACAGACCAGCTGCGGAAGATTTCCAAGGATGCGGGGATGCCTATCCAGGGTCAACCTTGTTTCTGCAAATATGCACAGGGGGCAGACAGCGTGGAGCCTATGTTCCGGCATCTCAAGAACACCTACTCAGGGCTGCAGCTCATTATTGTCATCCTGCCAGGGAAGACGCCGGTGTATGCTGAGGTGAAACGTGTCGGAGATACACTCTTGGGAATGGCTACGCAGTGTGTGCAGGTGAAGAACGTGGTCAAGACCTCACCTCAGACTCTGTCCAACCTCTGCCTCAAGATCAATGTCAAACTTGGTGGCATTAACAACATCCTAGTCCCACACCAGCGCTCTGCCGTTTTTCAACAGCCAGTGATATTCCTGGGAGCAGATGTTACACACCCCCCAGCAGGGGATGGGAAAAAACCTTCTATCACAGCAGTGGTAGGCAGTATGGATGCCCACCCCAGCCGATACTGTGCTACTGTGCGGGTACAGCGACCACGGCAAGAGATCATTGAAGACTTGTCCTACATGGTGCGTGAGCTCCTCATCCAATTCTACAAGTCCACCCGTTTCAAGCCTACCCGCATCATCTTCTACCGAGATGGGGTGCCTGAAGGCCAGCTACCCCAGATACTCCACTATGAGCTACTGGCCATTCGTGATGCCTGCATCAAACTGGAAAAGGACTACCAGCCTGGGATCACTTATATTGTGGTGCAGAAACGCCATCACACCCGCCTTTTCTGTGCTGACAAGAATGAGCGAATTGGGAAGAGTGGTAACATCCCAGCTGGGACCACAGTGGACACCAACATCACCCACCCATTTGAGTTTGACTTCTATCTGTGCAGCCACGCAGGCATCCAGGGCACCAGCCGACCATCCCATTACTATGTTCTTTGGGATGACAACCGTTTCACAGCAGATGAGCTCCAGATCCTGACGTACCAGCTGTGCCACACTTACGTACGATGCACACGCTCTGTCTCTATCCCAGCACCTGCCTACTATGCCCGCCTGGTGGCTTTCCGGGCACGATACCACCTGGTGGACAAGGAGCATGACAGTGGAGAGGGGAGCCACATATCGGGGCAGAGCAATGGGCGGGACCCCCAGGCCCTGGCCAAAGCCGTGCAGGTTCACCAGGATACTCTGCGCACCATGTACTTCGCTAAGCTCAGAGTTGGCATTGACTACAAGGATGACGATGACAAGGATTACAAAGACGACGATGATAAGGACTATAAGGATGATGACGACAAATAAGAATTCCTGT

*AGO2:* GGCTTTTTTGTTAGACGAAGCTTGGGCTGCAGGTCGACTCTAGAGGATCCAACTTTGTGCCAACCGGTCGCCACCATGTACTCGGGAGCCGGCCCCGCACTTGCACCTCCTGCGCCGCCGCCCCCCATCCAAGGATATGCCTTCAAGCCTCCACCTAGACCCGACTTTGGGACCTCCGGGAGAACAATCAAATTACAGGCCAATTTCTTCGAAATGGACATCCCCAAAATTGACATCTATCATTATGAATTGGATATCAAGCCAGAGAAGTGCCCGAGGAGAGTTAACAGGGAAATCGTGGAACACATGGTCCAGCACTTTAAAACACAGATCTTTGGGGATCGGAAGCCCGTGTTTGACGGCAGGAAGAATCTATACACAGCCATGCCCCTTCCGATTGGGAGGGACAAGGTGGAGCTGGAGGTCACGCTGCCAGGAGAAGGCAAGGATCGCATCTTCAAGGTGTCCATCAAGTGGGTGTCCTGCGTGAGCTTGCAGGCGTTACACGATGCACTTTCAGGGCGGCTGCCCAGCGTCCCTTTTGAGACGATCCAGGCCCTGGACGTGGTCATGAGGCACTTGCCATCCATGAGGTACACCCCCGTGGGCCGCTCCTTCTTCACCGCGTCCGAAGGCTGCTCTAACCCTCTTGGCGGGGGCCGAGAAGTGTGGTTTGGCTTCCATCAGTCCGTCCGGCCTTCTCTCTGGAAAATGATGCTGAATATTGATGTGTCAGCAACAGCGTTTTACAAGGCACAGCCAGTAATCGAGTTTGTTTGTGAAGTTTTGGATTTTAAAAGTATTGAAGAACAACAAAAACCTCTGACAGATTCCCAAAGGGTAAAGTTTACCAAAGAAATTAAAGGTCTAAAGGTGGAGATAACGCACTGTGGGCAGATGAAGAGGAAGTACCGTGTCTGCAATGTGACCCGGCGGCCCGCCAGTCACCAAACATTCCCGCTGCAGCAGGAGAGCGGGCAGACGGTGGAGTGCACGGTGGCCCAGTATTTCAAGGACAGGCACAAGTTGGTTCTGCGCTACCCCCACCTCCCATGTTTACAAGTCGGACAGGAGCAGAAACACACCTACCTTCCCCTGGAGGTCTGTAACATTGTGGCAGGACAAAGATGTATTAAAAAATTAACGGACAATCAGACCTCAACCATGATCAGAGCGACTGCTAGGTCGGCGCCCGATCGGCAAGAAGAGATTAGCAAATTGATGCGAAGTGCAAGTTTCAACACAGATCCATACGTCCGTGAATTTGGAATCATGGTCAAAGATGAGATGACAGACGTGACTGGGCGGGTGCTGCAGCCGCCCTCCATCCTCTACGGGGGCAGGAATAAAGCTATTGCGACCCCTGTCCAGGGCGTCTGGGACATGCGGAACAAGCAGTTCCACACGGGCATCGAGATCAAGGTGTGGGCCATTGCGTGCTTCGCCCCCCAGCGCCAGTGCACGGAAGTCCATCTGAAGTCCTTCACAGAGCAGCTCAGAAAGATCTCGAGAGACGCCGGCATGCCCATCCAGGGCCAGCCGTGCTTCTGCAAATACGCGCAGGGGGCGGACAGCGTGGAGCCCATGTTCCGGCACCTGAAGAACACGTATGCGGGCCTGCAGCTGGTGGTGGTCATCCTGCCCGGCAAGACGCCCGTGTACGCCGAGGTCAAGCGCGTGGGAGACACGGTGCTGGGGATGGCCACGCAGTGCGTGCAGATGAAGAACGTGCAGAGGACCACGCCACAGACCCTGTCCAACCTCTGCCTGAAGATCAACGTCAAGCTGGGAGGCGTGAACAACATCCTGCTGCCCCAGGGCAGGCCGCCGGTGTTCCAGCAGCCCGTCATCTTTCTGGGAGCAGACGTCACTCACCCCCCCGCCGGGGATGGGAAGAAGCCCTCCATTGCCGCCGTGGTGGGCAGCATGGACGCCCACCCCAATCGCTACTGCGCCACCGTGCGCGTGCAGCAGCACCGGCAGGAGATCATACAAGACCTGGCCGCCATGGTCCGCGAGCTCCTCATCCAGTTCTACAAGTCCACGCGCTTCAAGCCCACCCGCATCATCTTCTACCGCGACGGTGTCTCTGAAGGCCAGTTCCAGCAGGTTCTCCACCACGAGTTGCTGGCCATCCGTGAGGCCTGTATCAAGCTAGAAAAAGACTACCAGCCCGGGATCACCTTCATCGTGGTGCAGAAGAGGCACCACACCCGGCTCTTCTGCACTGACAAGAACGAGCGGGTTGGGAAAAGTGGAAACATTCCAGCAGGCACGACTGTGGACACGAAAATCACCCACCCCACCGAGTTCGACTTCTACCTGTGTAGTCACGCTGGCATCCAGGGGACAAGCAGGCCTTCGCACTATCACGTCCTCTGGGACGACAATCGTTTCTCCTCTGATGAGCTGCAGATCCTAACCTACCAGCTGTGTCACACCTACGTGCGCTGCACACGCTCCGTGTCCATCCCAGCGCCAGCATACTACGCTCACCTGGTGGCCTTCCGGGCCAGGTACCACCTGGTGGATAAGGAACATGACAGTGCTGAAGGAAGCCATACCTCTGGGCAGAGTAACGGGCGAGACCACCAAGCACTGGCCAAGGCGGTCCAGGTTCACCAAGACACTCTGCGCACCATGTACTTTGCTAAGCTCAGAGTTGGCATTGACTACAAGGATGACGATGACAAGGATTACAAAGACGACGATGATAAGGACTATAAGGATGATGACGACAAATAAGAATTCCTGTGGAATGTGTGTCAGTTAGGGTGTGGAAAGTCCCCAGGCTCCCCAGCAGGCAGAAGTATGCAAAGCATGCATCTCAATTAGTCAGCAACCAGGTGTGGAAA

*AGO3:* GACTCTAGAGGATCCAACTTTGTGCCAACCGGTCGCCACCATGGAAATCGGCTCCGCAGGACCCGCTGGGGCCCAGCCCCTACTCATGGTGCCCAGAAGACCTGGCTATGGCACCATGGGCAAACCCATTAAACTGCTGGCTAACTGTTTTCAAGTTGAAATCCCAAAGATTGATGTCTACCTCTATGAGGTAGATATTAAACCAGACAAGTGTCCTAGGAGAGTGAACAGGGAGGTGGTTGACTCAATGGTTCAGCATTTTAAAGTAACTATATTTGGAGACCGTAGACCAGTTTATGATGGAAAAAGAAGTCTTTACACCGCCAATCCACTTCCTGTGGCAACTACAGGGGTAGATTTAGACGTTACTTTACCTGGGGAAGGTGGAAAAGATCGACCTTTCAAGGTGTCAATCAAATTTGTCTCTCGGGTGAGTTGGCACCTACTGCATGAAGTACTGACAGGACGGACCTTGCCTGAGCCACTGGAATTAGACAAGCCAATCAGCACTAACCCTGTCCATGCCGTTGATGTGGTGCTACGACATCTGCCCTCCATGAAATACACACCTGTGGGGCGTTCATTTTTCTCCGCTCCAGAAGGATATGACCACCCTCTGGGAGGGGGCAGGGAAGTGTGGTTTGGATTCCATCAGTCTGTTCGGCCTGCCATGTGGAAAATGATGCTTAATATCGATGTTTCTGCCACTGCCTTCTACAAAGCACAACCTGTAATTCAGTTCATGTGTGAAGTTCTTGATATTCATAATATTGATGAGCAACCAAGACCTCTGACTGATTCTCATCGGGTAAAATTCACCAAAGAGATAAAAGGTTTGAAGGTTGAAGTGACTCATTGTGGAACAATGAGACGGAAATACCGTGTTTGTAATGTAACAAGGAGGCCTGCCAGTCATCAAACCTTTCCTTTACAGTTAGAAAACGGCCAAACTGTGGAGAGAACAGTAGCGCAGTATTTCAGAGAAAAGTATACTCTTCAGCTGAAGTACCCGCACCTTCCCTGTCTGCAAGTCGGGCAGGAACAGAAACACACCTACCTGCCACTAGAAGTCTGTAATATTGTGGCAGGGCAACGATGTATCAAGAAGCTAACAGACAATCAGACTTCCACTATGATCAAGGCAACAGCAAGATCTGCACCAGATAGACAAGAGGAAATTAGCAGATTGGTAAGAAGTGCAAATTATGAAACAGATCCATTTGTTCAGGAGTTTCAATTTAAAGTTCGGGATGAAATGGCTCATGTAACTGGACGCGTACTTCCAGCACCTATGCTCCAGTATGGAGGACGGAATCGGACAGTAGCAACACCGAGCCATGGAGTATGGGACATGCGAGGGAAACAATTCCACACAGGAGTTGAAATCAAAATGTGGGCTATCGCTTGTTTTGCCACACAGAGGCAGTGCAGAGAAGAAATATTGAAGGGTTTCACAGACCAGCTGCGTAAGATTTCTAAGGATGCAGGGATGCCCATCCAGGGCCAGCCATGCTTCTGCAAATATGCACAGGGGGCAGACAGCGTAGAGCCCATGTTCCGGCATCTCAAGAACACATATTCTGGCCTACAGCTTATTATCGTCATCCTGCCGGGGAAGACACCAGTGTATGCGGAAGTGAAACGTGTAGGAGACACACTTTTGGGTATGGCTACACAATGTGTTCAAGTCAAGAATGTAATAAAAACATCTCCTCAAACTCTGTCAAACTTGTGCCTAAAGATAAATGTTAAACTCGGAGGGATCAATAATATTCTTGTACCTCATCAAAGACCTTCTGTGTTCCAGCAACCAGTGATCTTTTTGGGAGCCGATGTCACTCATCCACCTGCTGGTGATGGAAAGAAGCCTTCTATTGCTGCTGTTGTAGGTAGTATGGATGCACACCCAAGCAGATACTGTGCCACAGTAAGAGTTCAGAGACCCCGACAGGAGATCATCCAGGACTTGGCCTCCATGGTCCGGGAACTTCTTATTCAATTTTATAAGTCAACTCGGTTCAAGCCTACTCGTATCATCTTTTATCGGGATGGTGTTTCAGAGGGGCAGTTTAGGCAGGTATTATATTATGAACTACTAGCAATTCGAGAAGCCTGCATCAGTTTGGAGAAAGACTATCAACCTGGAATAACCTACATTGTAGTTCAGAAGAGACATCACACTCGATTATTTTGTGCTGATAGGACAGAAAGGGTTGGAAGAAGTGGCAATATCCCAGCTGGAACAACAGTTGATACAGACATTACACACCCATATGAGTTCGATTTTTACCTCTGTAGCCATGCTGGAATACAGGGTACCAGTCGTCCTTCACACTATCATGTTTTATGGGATGATAACTGCTTTACTGCAGATGAACTTCAGCTGCTAACTTACCAGCTCTGCCACACTTACGTACGCTGTACACGATCTGTTTCTATACCTGCACCAGCGTATTATGCTCACCTGGTAGCATTTAGAGCCAGATATCATCTTGTGGACAAAGAACATGACAGTGCTGAAGGAAGTCACGTTTCAGGACAAAGCAATGGGCGAGATCCACAAGCTCTTGCCAAGGCTGTACAGATTCACCAAGATACCTTACGCACAATGTACTTCGCTAAGCTCAGAGTTGGCATTGACTACAAGGATGACGATGACAAGGATTACAAAGACGACGATGATAAGGACTATAAGGATGATGACGACAAATAAGAATTCCTGTGGAATGTGTGTCAGTTAGGGTGTGGAAAGTCCCCAGGCTCCCCAGCAGGCAGAAGTATGCAAAGCATGCATCTCAATTAGTCAGCAACCAGGTGTGGAAAGTCCCCAGGCTCCCCAGCAGGCAGAAGTATGCAAAGCATGCATCTCAATTAGTCAGCAACCATAGTCCCGCCCCTAACTCCGCCCATCCCGCCCCTAACTCCGCCCAGTTCCGCCCATTCTCCGCCCCATGGCTGACTAATTT
